# Hyper BOLD Activation in Dorsal Raphe Nucleus of APP/PS1 Alzheimer’s Disease Mouse during Reward-Oriented Drinking Test under Thirsty Conditions

**DOI:** 10.1038/s41598-020-60894-7

**Published:** 2020-03-03

**Authors:** Keisuke Sakurai, Teppei Shintani, Naohiro Jomura, Takeshi Matsuda, Akira Sumiyoshi, Tatsuhiro Hisatsune

**Affiliations:** 1 Department of Integrated Biosciences, Graduate School of Frontier Sciences, The University of Tokyo, Kashiwa, Japan; 2 National Institute of Radiological Sciences, National Institutes for Quantum and Radiological Science and Technology, Chiba, Chiba, 263-8555 Japan

**Keywords:** Alzheimer's disease, Alzheimer's disease

## Abstract

Alzheimer’s disease (AD), a neurodegenerative disease, causes behavioural abnormalities such as disinhibition, impulsivity, and hyperphagia. Preclinical studies using AD model mice have investigated these phenotypes by measuring brain activity in awake, behaving mice. In this study, we monitored the behavioural alterations of impulsivity and hyperphagia in middle-aged AD model mice. As a behavioural readout, we trained the mice to accept a water-reward under thirsty conditions. To analyse brain activity, we developed a measure for licking behaviour combined with visualisation of whole brain activity using awake fMRI. In a water-reward learning task, the AD model mice showed significant hyperactivity of the dorsal raphe nucleus in thirsty conditions. In summary, we successfully visualised altered brain activity in AD model mice during reward-oriented behaviour for the first time using awake fMRI. This may help in understanding the causes of behavioural alterations in AD patients.

## Introduction

Fifty million people worldwide currently suffer from dementia. This number is estimated to reach 130 million by 2050. Alzheimer’s disease (AD) is a neurodegenerative disease accounting for 60–70% of dementia cases^1^. AD develops through amnestic mild cognitive impairment (MCI), in which a decrease of memory function is the only symptom can be recognised. Once dementia develops, memory and various cognitive functions decrease, behavioural and psychological symptoms of dementia (BPSP) are also exhibited^1–3^. As these symptoms progress, it becomes impossible to maintain an independent daily life. Dementia patients mostly need medical care due to BPSP rather than reducing of memory and cognitive functions. BPSP includes depression, anxiety, and apathy. Although research on these BPSP symptoms has progressed, however, most symptoms such as disinhibition, impulsivity and hyperphagia have not been analysed in the way of pathology.

AD significantly impairs reward-based behaviour (by 15–20%) in patients. A typical example is hyperphagia, which stems from a lack of satiety control, similar to disorders of impulsivity and compulsivity^4,5^. The Urgency, lack of Perseverance, lack of Premeditation, Sensation Seeking (UPPS) Scale or Barrat’s Impulsiveness Scale (BIS) are now used to test for impulsivity in AD patients^3^. Overeating and impulsivity have also recently been reported in AD model mice^6,7^. These observations suggest that AD mice models may be useful in studying hyperphagia and early changes in impulsive behaviour. It has also been suggested that the brainstem serotonergic system, including the dorsal raphe nucleus^8,9^ and ventral temporal lobe (hippocampus, parahippocampus, amygdala), are involved in such alterations. However, the neurological mechanisms behind these phenotypes have not yet been identified. In order to elucidate these mechanistic underpinnings, direct evaluation of brain activity associated with abnormal behaviour phenotypes is required^10^.

Functional magnetic resonance imaging (fMRI) is widely used in neuroscience research to annotate changes in brain activity as effective biomarkers. Since fMRI is non-invasive, it can be used for early detection of AD and delaying onset of the disease. Though, fMRI has been used to analyse changes in brain activity related to BPSP in AD, we need to develop appropriate tasks that can be performed during the scan, which can allow meaningful inferences to be made. In human AD studies, fMRI has been performed both in resting state (rs-fMRI) and during task performance^11–14^. In AD model mice, however, studies are limited to rs-fMRI or anesthetised mice^10^. To our knowledge, no published studies exist in which AD model mice have performed tasks during fMRI. Though, the difference in the functional connectivity between the AD and control mice has been obtained, under these anesthetised conditions, it might not be possible to analyse brain activity during BPSP-associated animal behaviours. In order to evaluate brain activity of AD model mice during reward-oriented behaviour, in this study, we developed an experimental system for measuring brain activity by fMRI during licking behaviour under thirsty conditions. To identify the neurological mechanisms underlying BPSP in AD, imaging was performed using ultra-high field MRI (14 T) on mice to ensure the best possible trade-off for small animals^15^.

## Results

### Reward-oriented drinking behaviour to test impulsivity of AD model mice

**Figure 1:**
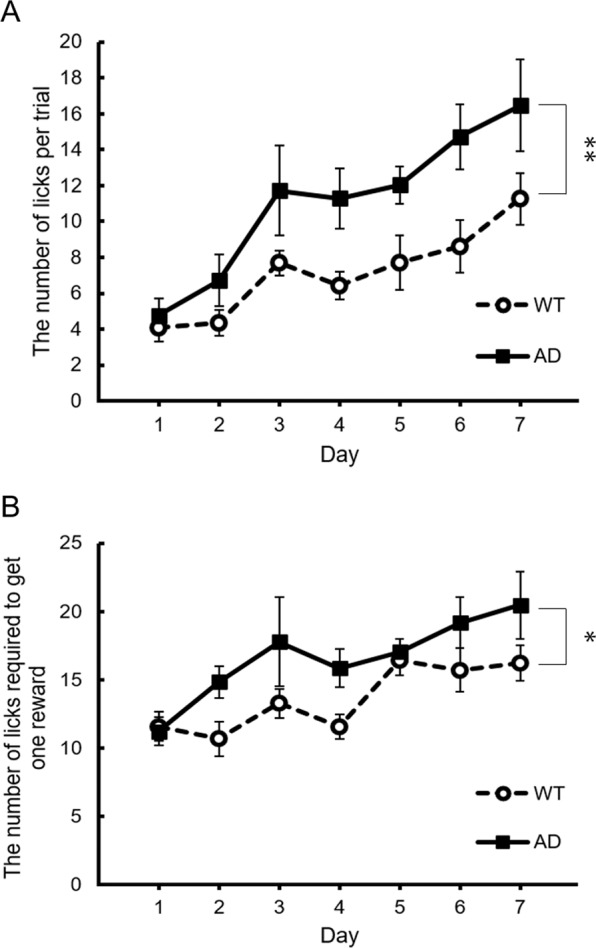
Licking behaviour during reward-oriented drinking test. (**A**) The number of licks per trials was significantly larger in AD model mice than in WT mice. (F[1.24] = 13.1237; p = 0.001359). (**B**) The number of licks required to get one reward was significantly larger in AD model mice than in WT mice. (F[1.24] = 5.756; p = 0.02455). Data are represented as mean ± SEM; ^*^p < 0.05, ^**^p < 0.01; two-way repeated measures ANOVA.

We developed a paradigm to measure impulsivity in mice, based on an operant learning system that can be applied to awake fMRI (Supplementary Fig. 1). Our designed behavioural experiments comprise four phases: surgery, 3 days of rest, 3 days of licking training, 7 days of cue-reward learning task. Three days after surgery, the water intake of mice was restricted (2 mL/day). The mice undergone licking training for 3 days were performed to leading habituation. Water was restricted by removing water bottles from breeding cages, and only providing water during the behavioural experiment, and compensating deficient water with a small dish containing up to 2 mL/day in individual cages. During the training task, mice were supplied water (4 µL/lick) through a nozzle at every time the sensor detected the licking action of their tongues. All training tasks were conducted until every mouse ingested water for 250 times. After the 3 days of training, mice were performed a cue-reward learning task (Supplementary Fig. 2) for 7 days. During the learning task, mice were also provided water (4 µL/correct licking action) through a nozzle only when they stuck out their tongues within 2 s of the last light stimulus. The light stimulus was given to both eyes from two light bulbs which placed in front of their faces. In the learning task, a series of 300 trials were performed and the number of licking actions was automatically measured. We delivered the conditioned stimulus (CS) using light and monitored the conditioned response (CR) using an optic sensor. Upon observing the CR, we delivered a water-reward. Mice were immobilised using a plastic head-bar and trained to drink water from a plastic nozzle. After this training period, each mouse performed the cue-reward learning task for 7 days. When we tested AD mice under this paradigm, the number of licks per trial was significantly higher for the AD mice than those for the wild-type (WT) mice (Fig. 1A; **p < 0.01). Based on post hoc analysis, the number of licks per trial was significantly higher in AD model mice on all of the day except the first. The number of licks required to obtain one reward was also significantly higher in AD model mice, suggesting elevated impulsivity (Fig. 1B; *p < 0.05).

### Awake fMRI during reward-oriented drinking behaviour

**Figure 2:**
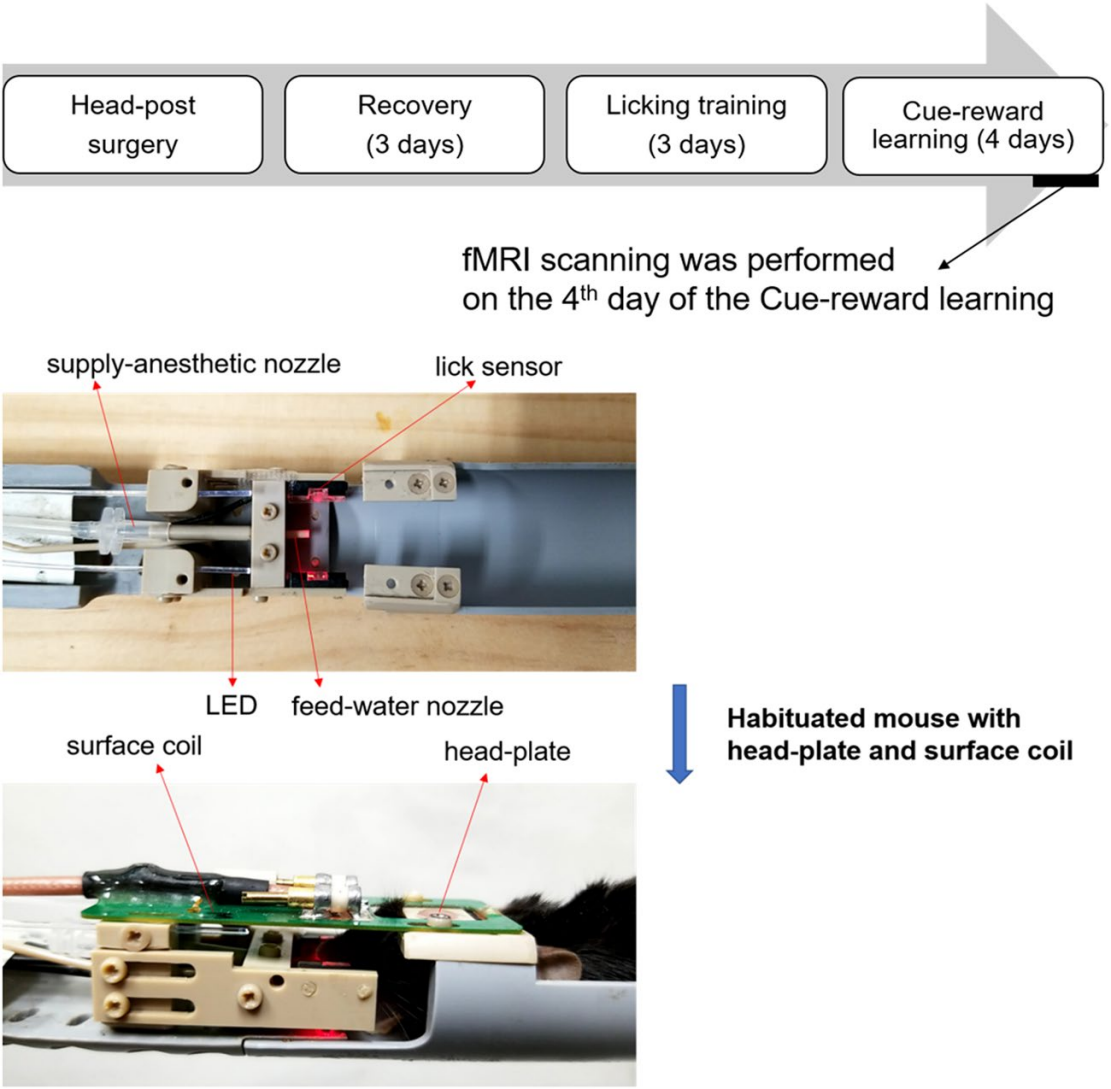
Schedule of reward-oriented drinking test and experimental device for fMRI. The reward-oriented drinking test for fMRI had four phases: head-post surgery, 3 days of recovery, 3 days of licking training, and 4 days of cue-reward learning task. fMRI scanning was performed on the fourth day of cue-reward learning task. Using habituated mice with a plastic head-bar (0.2 g) enabled minimisation of head movement.

For analysis of brain activity in AD model mice, we designed an experimental scheme to obtain fMRI from behaving mice (Fig. 2). A non-magnetic head-fixed device and fMRI were used to measure brain activity during licking behaviour under thirsty conditions. Mice were stimulated with light and licking behaviour as CR was detected using a sensor. If a CR was detected, 4 μL of water reward was given to the mouse. Prior to fMRI, we performed light stimulation on the left eye of anesthetised WT mice (n = 3) and analysed activation of the right primary visual cortex to confirm detection of cerebral activation. The stimulus consisted of a blinking light for 10 s, which was then turned off for 10 s. This on-off pattern was repeated 15 times. The anesthesia level was kept at 2% isoflurane in oxygen-enriched air (~30% O_2_) during the experiment. The blood-oxygen-level-dependent (BOLD) activation of control mice and its time course in the visual cortex after light stimulation is shown in Supplementary Fig. 3. As seen in the histogram, elevation of BOLD signal was detected using our system.

The tasks performed in the MRI scanner are the same as the tasks performed in advance for behaviour analysis, and consist of four phases: surgery, rest, licking training, and cue-reward association learning. Additionally, for habituation to fMRI scanning, we made mice listen to recorded fMRI scanning sounds during the licking training phase and also made them perform the learning task inside the machine. In order to evaluate activation of the reward system, including ventral and dorsal striatum, in awake behaving mice, we statistically analysed the difference in BOLD signals before and after sequential post-processing. Analysis revealed that the BOLD signals were elevated in the reward systems (Supplementary Fig. 4; *p* < 0.05 FWE-corrected at peak level).

### Awake fMRI from AD model mice during reward-oriented drinking behaviour

**Figure 3:**
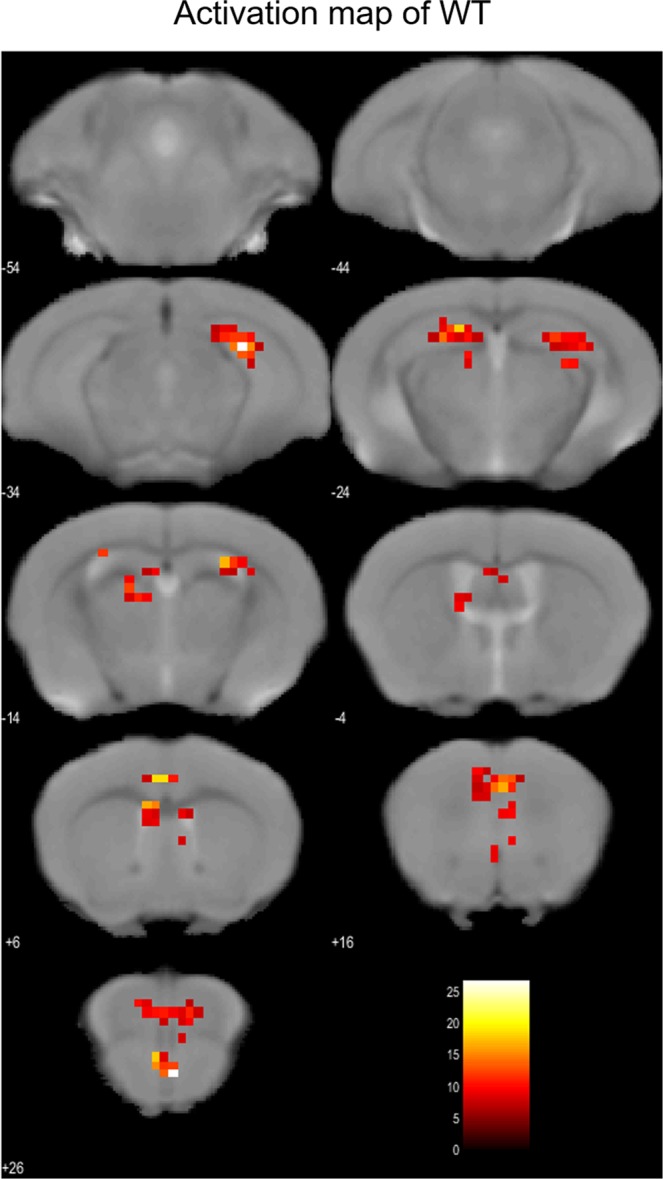
Activated areas in WT mice during reward acquisition. In WT mice (n = 3; male), activation was observed in orbitofrontal, medial prefrontal, and cingulate cortices, striatum, and hippocampus, but not in parahippocampal gyrus and raphe nucleus. Images were drawn by *p* < 0.001 (uncorrected at voxel level), threshold k > 10, scale bar represents T-score. At the cluster-level, *p* < 0.001 _FWE_−_corr_.

**Figure 4:**
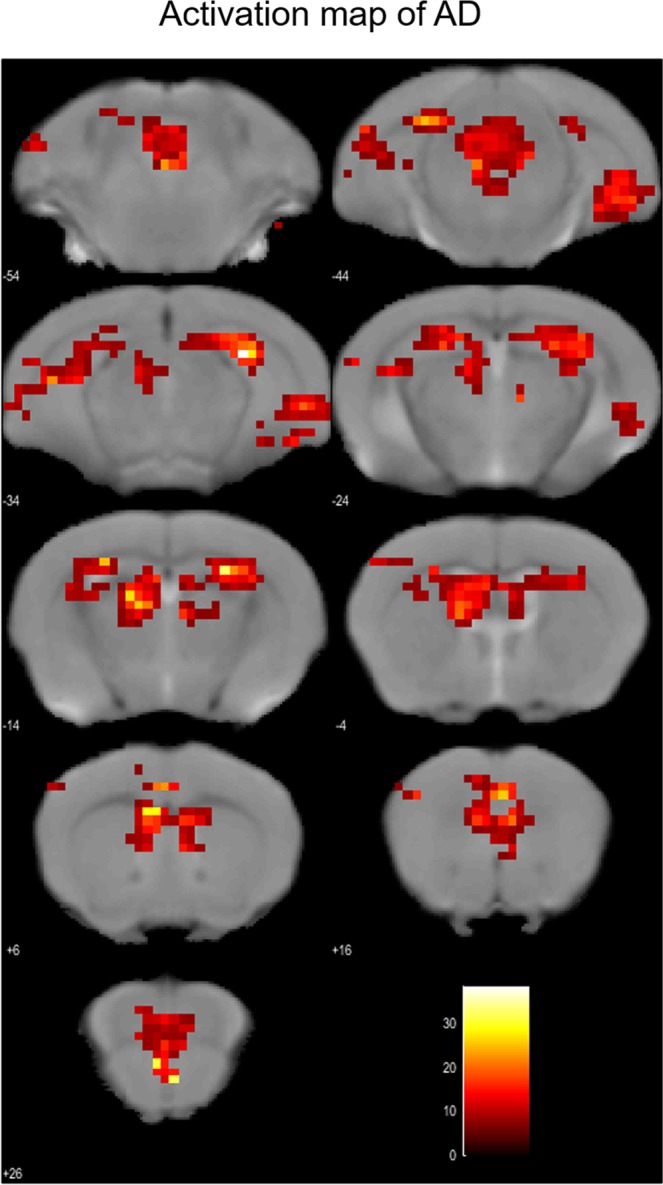
Activated areas in AD model mice during reward acquisition. In AD model mice (n = 3, male), activation was observed in orbitofrontal, medial prefrontal, and cingulate cortices, striatum, hippocampal formation and raphe nucleus. Images were drawn by <0.001 (uncorrected at voxel level), threshold k > 10, scale bar represents T-score. At the cluster-level, *p* < 0.001 _FWE_−_corr_.

**Figure 5:**
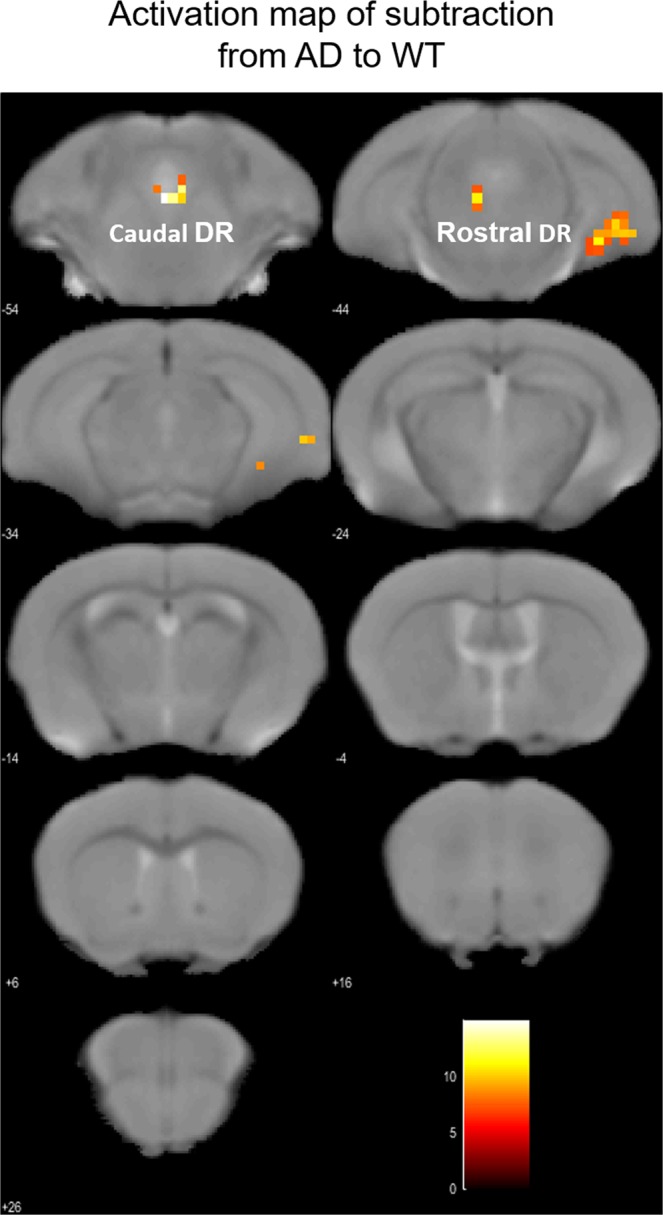
Areas in AD mice that were significantly more activated than in WT mice. Hippocampus, subiculum, entorhinal cortex, dentate gyrus, and dorsal raphe were significantly more activated in AD mice than in WT mice during reward acquisition. Images were drawn by *p* < 0.001 (uncorrected at voxel level), threshold k > 10, scale bar represents T-score. At the cluster-level, *p* < 0.001 _FWE_−_corr_.

The above set-up was used to analyse brain activity in awake, behaving AD model mice. As a result of second level analysis, in both the WT and AD model mice, we detected areas where brain activity was significantly increased (Figs. 3 and 4). For this analysis, *p* < 0.001 (uncorrected at peak level) and *p* < 0.001 (FWE-corrected at cluster-level) were defined as significance thresholds. A wider range of brain regions was activated in AD model mice, and robust activation was observed in the dorsal raphe nucleus and the hippocampal formation (Fig. 4). On the other hands, in WT mice, we just observed the activation of the dorsal hippocampus and no activation in the dorsal raphe nucleus (Fig. 3). To identify features specific to AD mice, brain areas of AD mice that were more active than in the WT group were tested with a two-sample t-test. Elevated BOLD activation was seen in the dorsal raphe nucleus and ventral hippocampus in AD models, when compared to control mice (Fig. 5). There were no brain areas in control mice that were more significantly activated than in AD models. In the correct trials, the average time of sticking out the tongue after the initiation of the light stimuli was in AD mice as 536 ± 25 ms (average ± SD), and in WT mice as 909 ± 364 ms. There was no difference between the two groups (*p* = 0.22). The reward was presented 2 second after the initiation of the light stimuli.

**Figure 6:**
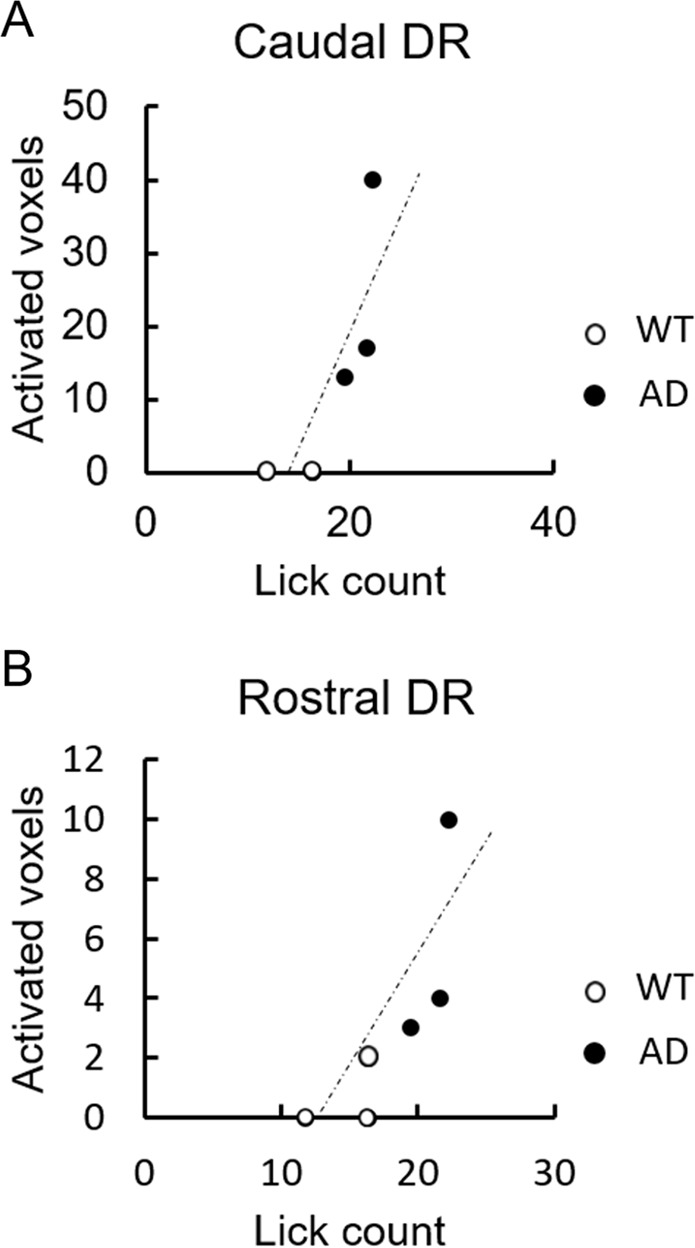
Correlation between the impulsive behaviour and the hyperactivity in dorsal raphe nucleus. The impulsive behaviour in rewarded trial was evaluated by the licking counts after the conditioning stimuli (CS) in AD (n = 3; closed circle) and WT (n = 3; open circle) mice, of which fMRI data described in Figs. 3–5. The average number of rewarded trails in total 15 sessions along with fMRI scanning was in AD mice as 12.7 ± 1.2 (average ± SD), and in WT mice as 8.7 ± 1.5 (*p* = 0.022, student’s t-test, between the two group), suggesting elevated impulsivity in AD as shown in Fig. 1. The hyperactivity was evaluated by significantly activated voxels in caudal or rostral dorsal raphe nucleus in each mouse after the end of CR (0–2 s), in comparison with the baseline (FWE correction, *p* < 0.05). Correlation was analysed by the Pearson correlation coefficient. In caudal dorsal raphe (R = 0.814, *p* = 0.0488; **A**) and in rostral dorsal raphe (R = 0.803, *p* = 0.0547; **B**).

Based on our hypothesis of a link between impulsive behaviours and dorsal raphe nucleus, we set ROI (region of interest) on caudal dorsal raphe and rostral dorsal raphe and compared differences between groups of the number of activated voxels in the ROI by two sample t-test. As a result, AD mice had significantly more activated voxels in both the caudal dorsal raphe (**p = 0.006) and rostral dorsal raphe (*p = 0.017). Besides, to investigate the correlation between hyperactivity of dorsal raphe and impulsive behaviour, we calculated the correlation coefficient between the number of activation voxels in dorsal raphe and the average number of licks between the initiation of the light stimulation and 6 s after the end of the light stimulation in a correct trial. As a result, there were large correlations between the number of activated voxels and the number of licks when correct trial in both the caudal dorsal raphe (R = 0.81) and rostral dorsal raphe (R = 0.80) (Fig. 6).

**Figure 7:**
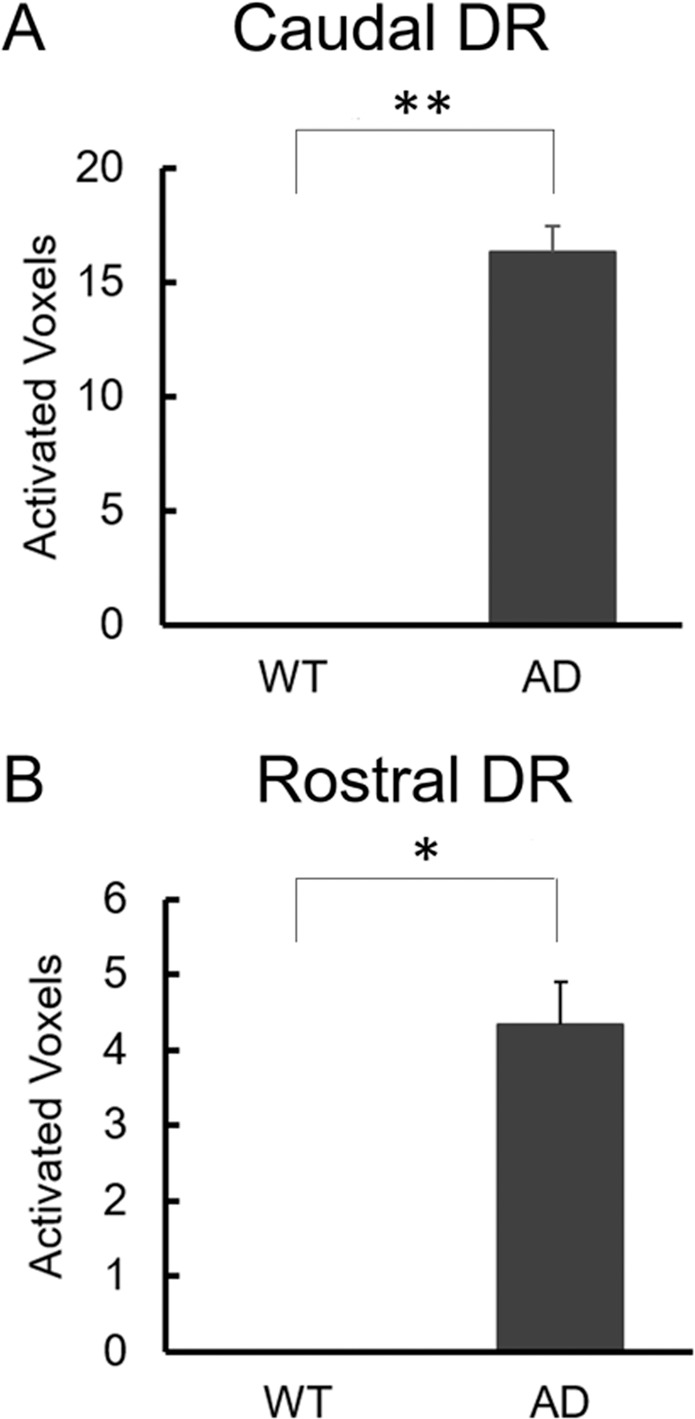
The number of activated voxels of caudal and rostral dorsal raphe of analyses including motor execution effect. (**A**) The number of activated voxels of caudal dorsal raphe was significantly larger in AD model mice (n = 3) than in WT mice (n = 3). (*p* = 0.0039, student’s t-test, between the two group). (**B**) The number of activated voxels of rostral dorsal raphe was significantly larger in AD model mice (n = 3) than in WT mice (n = 3). (*p* = 0.0121, student’s t-test, between the two group). Data are represented as mean ± SEM; ^*^p < 0.05, ^**^p < 0.01.

In addition, in order to confirm that the hyperactivation of caudal and dorsal raphe in AD mice was not due to motor execution, but to AD-related symptoms, we re-analysed by adding the confound regressor to GLM model. As a result, AD mice had significantly more activated voxels in both the caudal dorsal raphe (Fig. 7A; **p = 0.004) and rostral dorsal raphe (Fig. 7A; *p = 0.012), as with the analysis without confound regressor. On the other hand, there was no significantly activated voxel of dorsal raphe in all WT mice.

### Open field test

We performed the open field test to verify whether anxiety-like behaviour and reduced motivation were observed in AD model mice. In rodents, it is known that as anxiety increases, the willingness to explore threatening stimuli decreases, so exploratory behaviour decreases and they prefer to stay close to the walls of the field. As a result of open field test, the time spent inside the field and the total travel distance were not significantly different between the groups (Supplementary Fig. 5). Therefore, we assumed that there was no decrease in motivation or increased anxiety specific to AD mice.

## Discussion

In this study, we created an experimental system to analyse behavioural abnormalities in AD model mice, including increased impulsive and compulsive response of hyperphagia. During a reward-oriented drinking test, the number of licks per trial and those required to obtain a single reward were both higher in AD model mice than in WT mice, indicating elevated impulsivity in AD models. It has already been reported that some AD patients show altered impulsivity^3^.

The fMRI conducted in AD model mice during reward-oriented drinking tests showed abnormally high activation in the dorsal raphe nucleus and hippocampal formation. The hippocampal formation is one of the earliest and most prominently affected brain structures in AD patients, followed by degeneration of association cortices and the limbic system, including the amygdala^16^. In fMRI studies, hippocampal hyperactivity is observed in several conditions preceding AD, including MCI^17–22^, and has been reported to predict cognitive decline in MCI subjects^23,24^. Cognitive decline can be improved by administering a specific antiepileptic drug that suppresses hippocampal hyperactivity^25^. These reports contradict the general view that enhanced cerebral hemodynamics lead to functional improvement. Hippocampal hyperactivation in middle-aged AD model mice, as observed in this study, suggests that their brains mimic the cognitive dysfunction- associated activity in MCI patients.

The brainstem serotonergic system is required for controlling impulsiveness^26–29^. Inhibition of the dorsal raphe nucleus, the main serotonin source, alters impulsivity by reducing tolerance to delayed rewards and enhancing avoidance of difficulty selection^30,31^. As AD progresses, loss of serotonergic neurons and/or hyperphosphorylation of tau proteins accompany local neuroinflammation in the dorsal raphe^8,9,32,33^. Besides, as a result of re-analysis including a confound regressor (the number of motor response in each trial), hyperactivation of the dorsal raphe nucleus in AD model mice was observed as well. Therefore, we concluded that hyperactivation of the dorsal raphe nucleus in AD model mice observed in this study may reflect the enhanced impulsivity characteristic of AD, not greater motor execution.

In this study, we successfully used awake fMRI to visualise brain activity in AD model mice during task execution for the first time. Such methods, accompanied by opto- or chemogenetics, can further investigations in AD model mice. It is especially advantageous as similar manipulations cannot be performed on humans^34–36^. The ease of genetically manipulating mice makes them suitable for investigating the roles of specific genes in brain function. Events like amyloid plaque deposition or tau protein hyperphosphorylation can be individually examined using specific model mice. Therefore, fMRI studies on rodent AD models are effective in understanding AD progression. Up to now, such studies have mostly imaged mice under anesthesia or used rs-fMRI because it is difficult to suppress the noise due to body movement^37–39^. Anesthesia can suppress BOLD signals or report brain activity differently from what can be seen in awake animals^40–43^. The main disadvantage of these methods is their inability to associate brain activity with behaviour. Therefore, fMRI studies in awake animals are vital^44–47^. Very recently, Han *et al*. have nicely developed a protocol to perform awake and behaving mouse fMRI during Go/No-Go task and presented a brain-wide activation pattern of both Go and No-Go tasks^47^. Together with this innovative new protocol, our experimental system will offer promises to advance fundamental AD research and indispensable information to understand the etiology of AD.

## Methods

### Animals

We used B6C3-Tg (APPswe / PSEN1dE9) double transgenic mouse as AD models. Amyloid Precursor Protein (APP) and Presenilin1 (PSEN1) have been identified as causative genes for AD. They were expressed under independent mouse prion protein (PrP) promoters in their respective expression vectors as described before^48^. The mice were purchased from Jackson Laboratory (Bar Harbor, Maine, USA), self-mated in laboratory breeding rooms and used for experiments as described previously^49,50^. They were sorted by age and genotype, kept under a 12-hour light / dark cycle at ~22 ° C, and provided with enough food (solid feed MF; Oriental Yeast Co., Ltd., Tokyo, Japan) and water. We used 12-month-old APP/PS1 transgenic mice as AD models and the age-matched wild-type littermates as controls. All experiments were approved by the Ethics Committee of the University of Tokyo and conducted according to the institutional guidelines for animal experiments.

### Head fixation and cue-reward learning device for fMRI

We developed a non-magnetic head-fixed experimental system, manufactured by O’Hara & Co., Ltd. (Tokyo, Japan), for measuring brain activity by fMRI during licking behaviour under thirsty conditions (Fig. 2). In previous studies, head posts were attached by surgery^51,52^ before fMRI to suppress movement during scanning. In the Skinner box, we adopted light stimulus as cue and water as reward. We utilised an optic sensor to sense the licking action of the mouse tongue instead of a lever to minimise the influence caused by body movement, and confirmed that head motions during my fMRI session were suppressed (Supplementary Fig. 6).

### Surgery for attaching head plate

On the first day, we performed surgery to place a plate on the head of the mice for head immobilisation. The mice were deeply anesthetised by intramuscular injection of ketamine (50 mg/kg)/xylazine (5 mg/kg) and the centre of the plate was fitted to the mouse bregma, keeping their visual perception clear using a stereotactic device. After the surgery, the mice were kept in cages with food and water freely for 3 days to regain enough strength to perform the behavioural experiments.

### fMRI optimisation

The MRI apparatus used in this study has higher magnetic field (14 T) than conventional systems. fMRI studies on mice require higher spatial resolution due to their small size. Reducing voxel size increases spatial resolution, but also decreases BOLD signal per voxel and, hence, signal-to-noise ratio (SNR) as well^53^. Since higher magnetic field can enhance BOLD signals^54,55^, ultra-high field MRI has been used in some studies^39,56^. We used Spin Echo-Echo Planar Imaging (SE-EPI) sequence for high-resolution fMRI at ultra-high magnetic fields^55,57^. A custom-made surface coil for 14 T-MRI was attached to the operant learning device on top, and the coil centre was fitted to the mouse bregma. We compared scans with and without light stimulation using two-sample t-tests in SPM12 software (Wellcome Trust Center for Neuroimaging, UK) and confirmed the activation of the right primary visual cortex. We set the right primary visual cortex as the region of interest (ROI) and plotted changes in the BOLD signal. We confirmed that it peaked 8 s after the start of light stimulation and returned to the baseline 10 s after the end of light stimulation.

### Image acquisition during reward-oriented drinking test

We used habituated head-fixed mice to obtain BOLD fMRI data during cue-reward licking behaviour. Before task execution, structural MR images were acquired using a two-dimensional multi-slice T2-weighted fast spin echo sequence with the following parameters: echo time (TE)/(TR) = 30/2000 ms, flip angle = 90°, field of view (FOV) = 17 × 17 mm2, matrix size = 64 × 64, in-plane resolution = 0.269 mm × 0.269 mm, 6 slices with a slice thickness of 0.75 mm and number of averages = 8. We obtained a series of scans (105 fMRI; 15 trials × 7 scans per trial) during task execution. The data were acquired using SE-EPI with the following parameters: TE/TR = 30/2000 ms, flip angle = 90°, FOV = 17 × 17 mm2, matrix size = 64 × 64, in-plane resolution = 0.269 mm × 0.269 mm, 6 slices with a slice thickness of 0.75 mm. On the fourth day of learning, the whole brain, excluding the olfactory bulb and cerebellum, was scanned at the first 15 trials.

### Image processing and statistical analysis

Pre-processing and statistical analyses were performed using SPM12 software. We first spatially realigned images with reference to the first volume to correct for head movement and then corrected the time lag to match the middle slice. After that, in order to align brain functional and structural images, we calculated the average image of the EPI image after realignment processing and co-registered with the T2 image. All T2 images were processed with Advanced Normalisation Tools software^58^: (i) image was manually rotated and translated such that the origin of the coordinates occupied the midpoint of the anterior commissure to roughly match the standard reference space; (ii) single reference image was manually skull-stripped using “ITK-SNAP” software^59^ to create the brain mask; (iii) other subject images were registered to the reference image and skull-stripped; (vi) image non-uniformity was corrected and image intensity was normalised; (v) minimum deformation template (MDT) was constructed with the SyN nonlinear registration algorithm; (vi) the obtained MDT was manually skull-stripped and registered to the atlas space^60^; and (vii) the functional maps were also registered to the MDT space and the atlas space.

We smoothened the data with a Gaussian kernel with full width at half maximum (FWHM) of 0.8 mm to alleviate noise arising from pre-processing and individual differences in the brain. After pre-processing, in order to investigate brain activation area under reward-oriented behaviour of AD model mice and WT mice, we performed post-processing first-level analysis on data from individual animals using rewarded trials with the convolution of Canonical Hemodynamic Response Function (HRF) in SPM12. In the first-level analysis, we add 6 dimensional head movement parameters calculated at the realignment process as additional explanatory variables to GLM, and alleviated the effect of head movement on the BOLD signal. We utilised the series of fMRI data and calculated event-related BOLD activation 0–2 s after the end of 2 second CS, in comparison to the baseline BOLD signal before the CS (−2–0 second) with the convolution of HRF. After that, in order to make inferences about group data and estimate the specific differences in brain activation areas between AD and WT groups, we performed second-level analysis using the contrast created in the first level analysis. Here, the brain activation area in each group was confirmed by one-sample t-test. In order to identify the activation area during reward-oriented behaviour specific to the AD group, the brain area of the AD group that was more significantly activated than in the WT group was tested by two-sample t-test. For ROI-analysis, we utilised a custom ROI for the caudal dorsal raphe and rostral dorsal based on the mouse atlas by Franklin & Paxinos^61^, combined with the MRI-based digital atlases^60,62^.

In addition, in order to investigate the possibility that higher activity of dorsal raphe in AD mice is not due to AD-related symptoms, but due to greater motor execution, we re-conducted the first-level analyses including a confound regressor (the number of motor response in each trial) in the subject-level GLM model, and compared the differences between groups of activated voxel numbers of rostral and caudal dorsal raphe utilising the same ROI.

### Open field test

Open field test is developed by Calvin Hall to assess exploratory willingness and anxiety in animal models^63^. Rodents generally prefer to stay in the dark and close to walls, but they are also have the willingness to explore threating stimuli. Increasing anxiety reduces exploratory willingness, so that they travel less and spend less time inside the field^64^. In this experiment, we used an open field chamber of 45 cm × 45 cm × 30 cm. We defined 20 cm × 20 cm square area in the middle of the chamber as the inside of the field. For the open field experiment, we prepared AD mice (n = 10) and WT mice (n = 10). The mice were allowed to move freely in 10 minutes, and SMART video tracking software was used to measure total travelled distance and time spent inside of the field.

### Statistical analyses

In order to evaluate brain activity related to abnormal behaviour phenotype in AD model mice, we analysed the licking behaviour of mice during reward-oriented behaviour and obtained fMRI image from AD (n = 3) and WT (n = 3) male mice. For the licking behaviour analysis (Fig. 1), we prepared male AD model mice (n = 13) and WT mice (n = 13). Time-series behavioural data (Fig. 1) were analysed using two-way repeated measures ANOVA to test for overall effect of genotype and interaction between groups and sessions. Behavioural data of the open field test were analysed using student’s t-test.

## Supplementary information

Supplementary figures.

## Data Availability

Data can be obtained by contacting the corresponding author.
