# Supplementary material for: Hyper BOLD Activation in Dorsal Raphe Nucleus of APP/PS1 Alzheimer’s Disease Mouse during Reward-Oriented Drinking Test under Thirsty Conditions
Source: Sci Rep. 2020 Mar 3;10:3915. doi: 10.1038/s41598-020-60894-7 (PMC7054396; doi:10.1038/s41598-020-60894-7)
Supplement: Supplementary file 1 — Supplementary figures. [file 41598_2020_60894_MOESM1_ESM.pdf]

Hyper BOLD Activation in Dorsal Raphe Nucleus of APP/PS1 Alzheimer's Disease Mouse during Reward-Oriented Drinking Test under Thirsty Conditions

Keisuke Sakurai[1], Teppei Shintani[1], Naohiro Jomura[1], Takeshi Matsuda[1], Akira Sumiyoshi[2], and Tatsuhiro Hisatsune[1]*

[1]Department of Integrated Biosciences, Graduate School of Frontier Sciences, The University of Tokyo, Kashiwa, Japan

[2]National Institute of Radiological Sciences, National Institutes for Quantum and Radiological Science and Technology, Chiba, Chiba 263-8555, Japan

*Corresponding author: Tatsuhiro Hisatsune, PhD, Department of Integrated Biosciences, The University of Tokyo, Kashiwanoha 5-1-5, Biosciences Bldg., Room 402, Kashiwa, Chiba 277-8562, Japan. Tel.: + 81 4 7136 3632; Fax: + 81 4 7136 3633; E-mail: hisatsune@edu.k.u-tokyo.ac.jp.

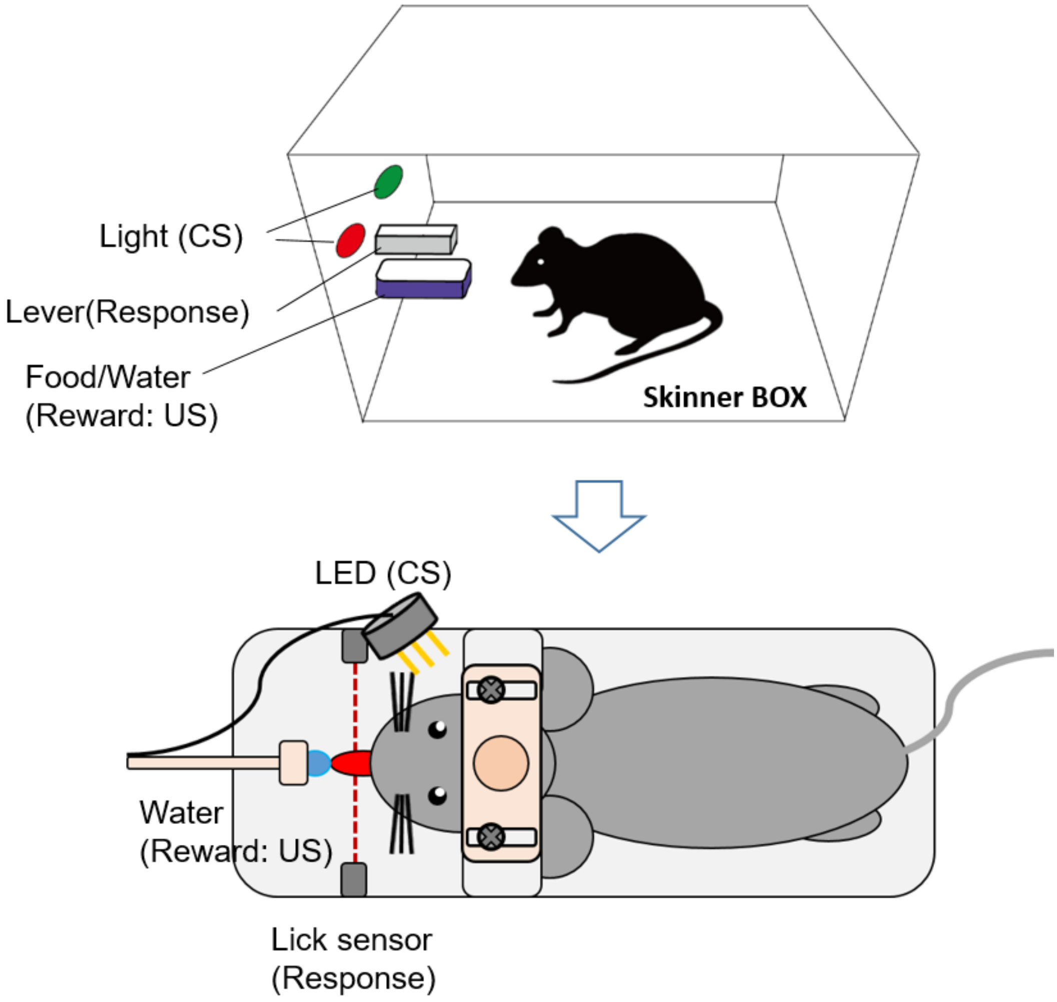

Supplementary Figure 1. Cue-reward learning device as a modified Skinner box
The light stimulator of the Skinner box corresponds to the LED light of this device. The lever corresponds to the lick sensor. The feed supply device corresponds to the water tube attached to the operating computer system (O'Hara and Co., Tokyo, Japan). The mice were immobilised using head plates and made to perform learning tasks.

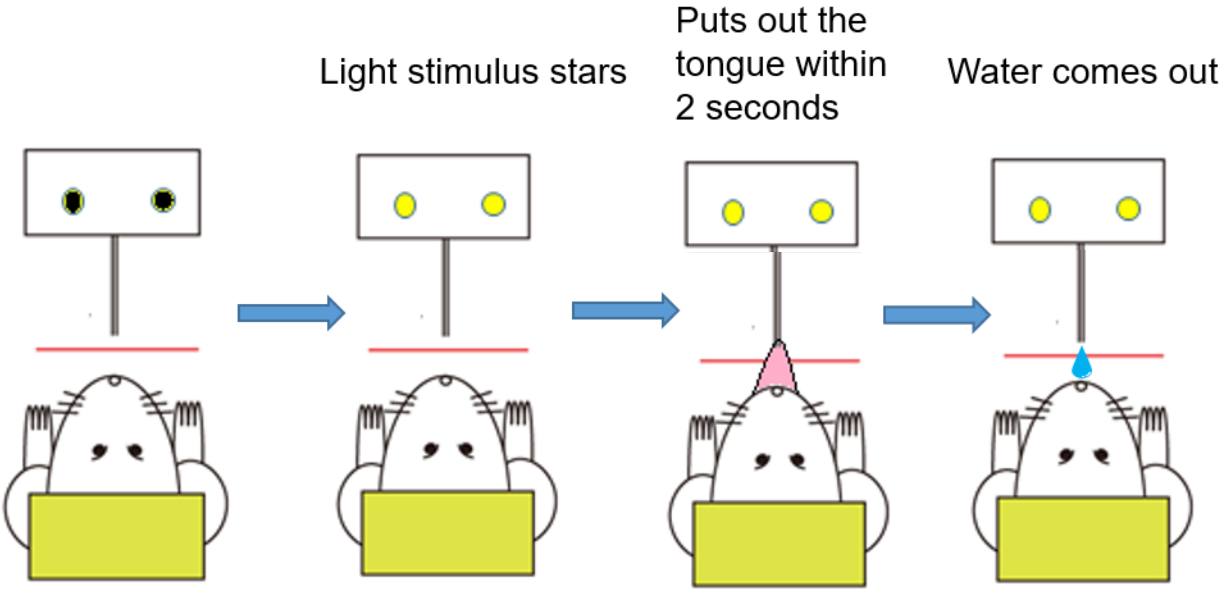

Supplementary Figure 2. Details of cue-reward learning task
In the cue-reward learning task, the mice were provided water (4 µL/correct licking action) through a water-supplying nozzle only when they stuck their tongues out づ during 2 s of the light stimulation. The light stimulation was given to both eyes of mice from two LED bulbs placed in front of their faces.

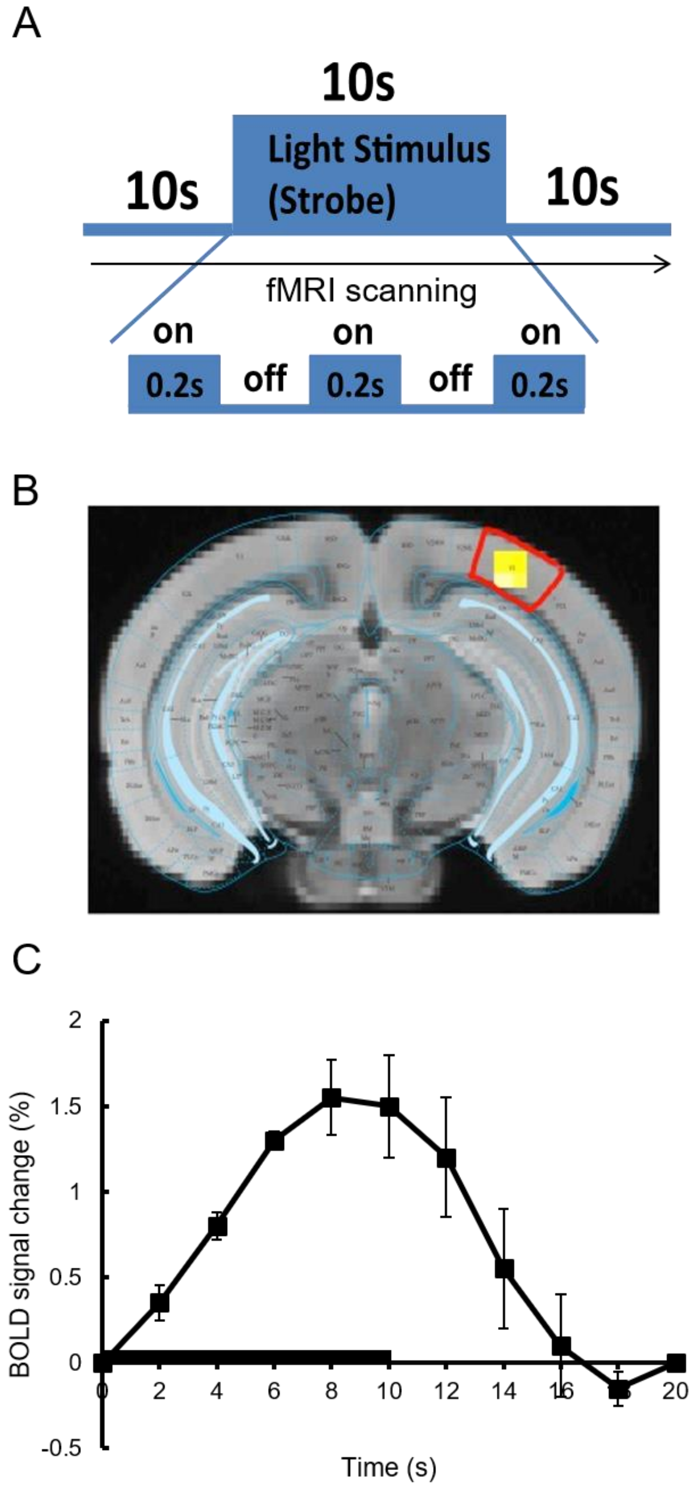

Supplementary Figure 3. BOLD response during visual stimulation
(A) Visual stimulation during fMRI consisted of a blinking light for 10 s, which was then turned off for 10 seconds. This on-off pattern was repeated 15 times.
(B) Upon light stimulation, the primary visual cortex was significantly activated when compared to the unstimulated condition. The red line frame indicates the primary visual cortex. $p < 0.05$ (FWE-corrected at cluster level) and $p < 0.001$ (uncorrected at peak level), threshold $k = 0$, scale bar represents T-score.
(C) We set ROI as the right primary visual cortex and plotted changes in the BOLD signal. We confirmed that it peaked 8 s after the start of light stimulation and returned to the baseline 10 s after the end of light stimulation.
The black bar indicates the time during which light stimulation was performed. Data are represented as mean ± SEM.

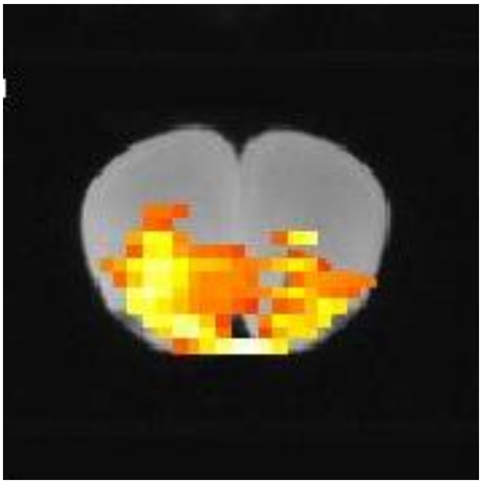

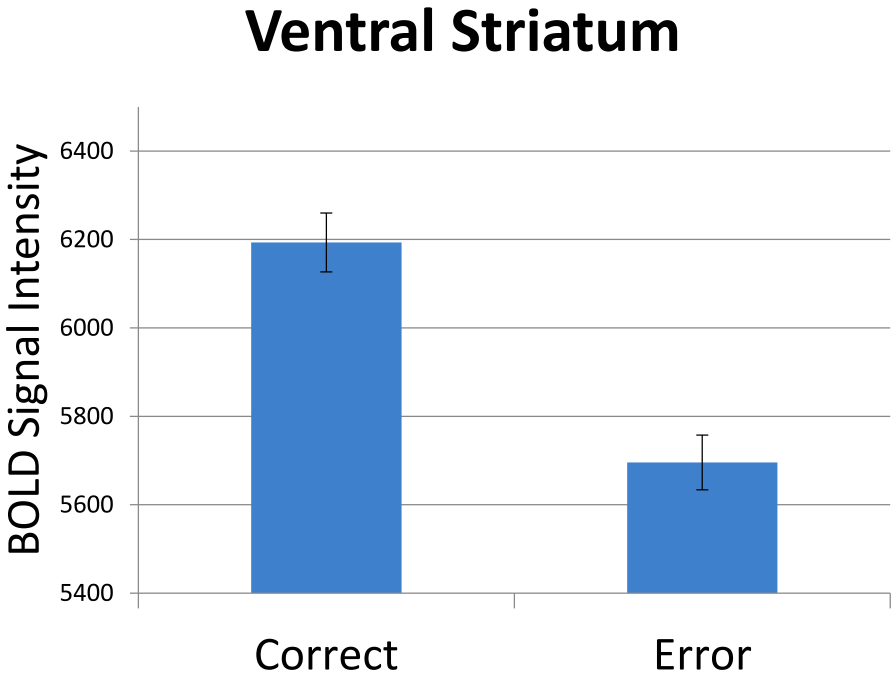

Correct – Error: ***P<0.001: Student's t-test

Supplementary Figure 4. BOLD response after light stimulation during fMRI in awake, behaving mice
In order to account for haemodynamics, we set each BOLD fMRI for 1.8 s in SE sequence, 2 s after initiation of the light stimulus.

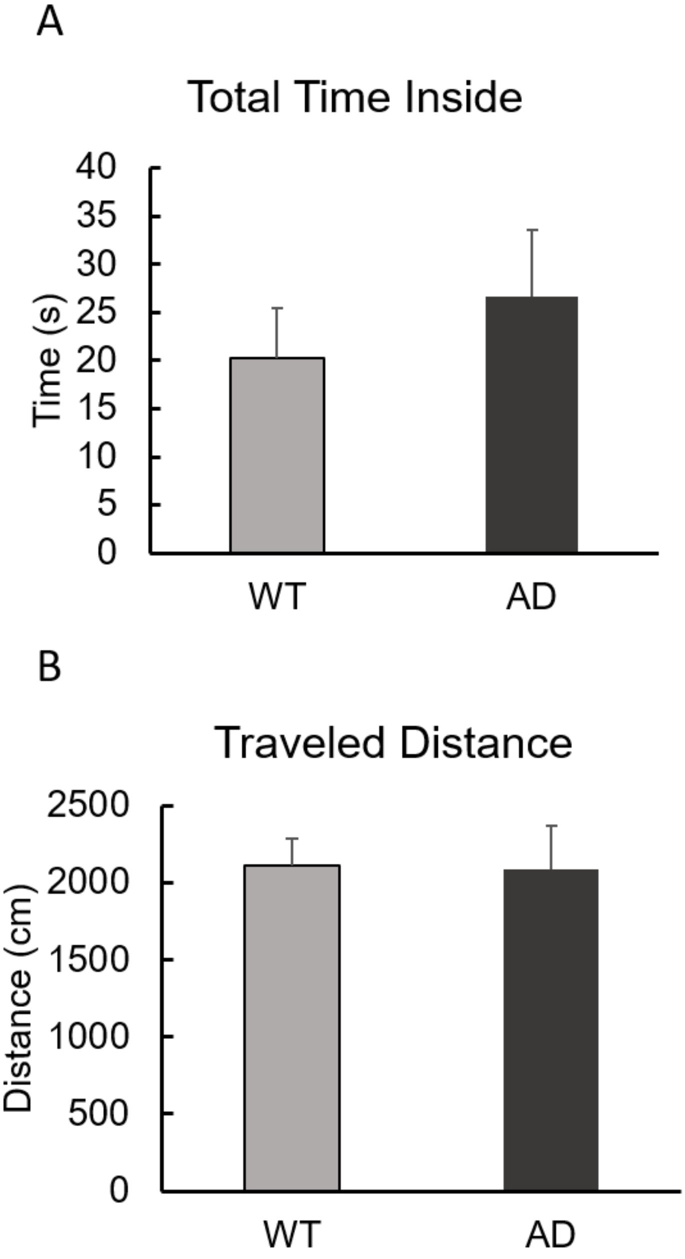

Supplementary Figure 5. Open field test
(A) The time spent inside the field was not significantly different between the groups (p = 0.46).
(B) The total travel distance was not significantly different between the groups (p = 0.93).

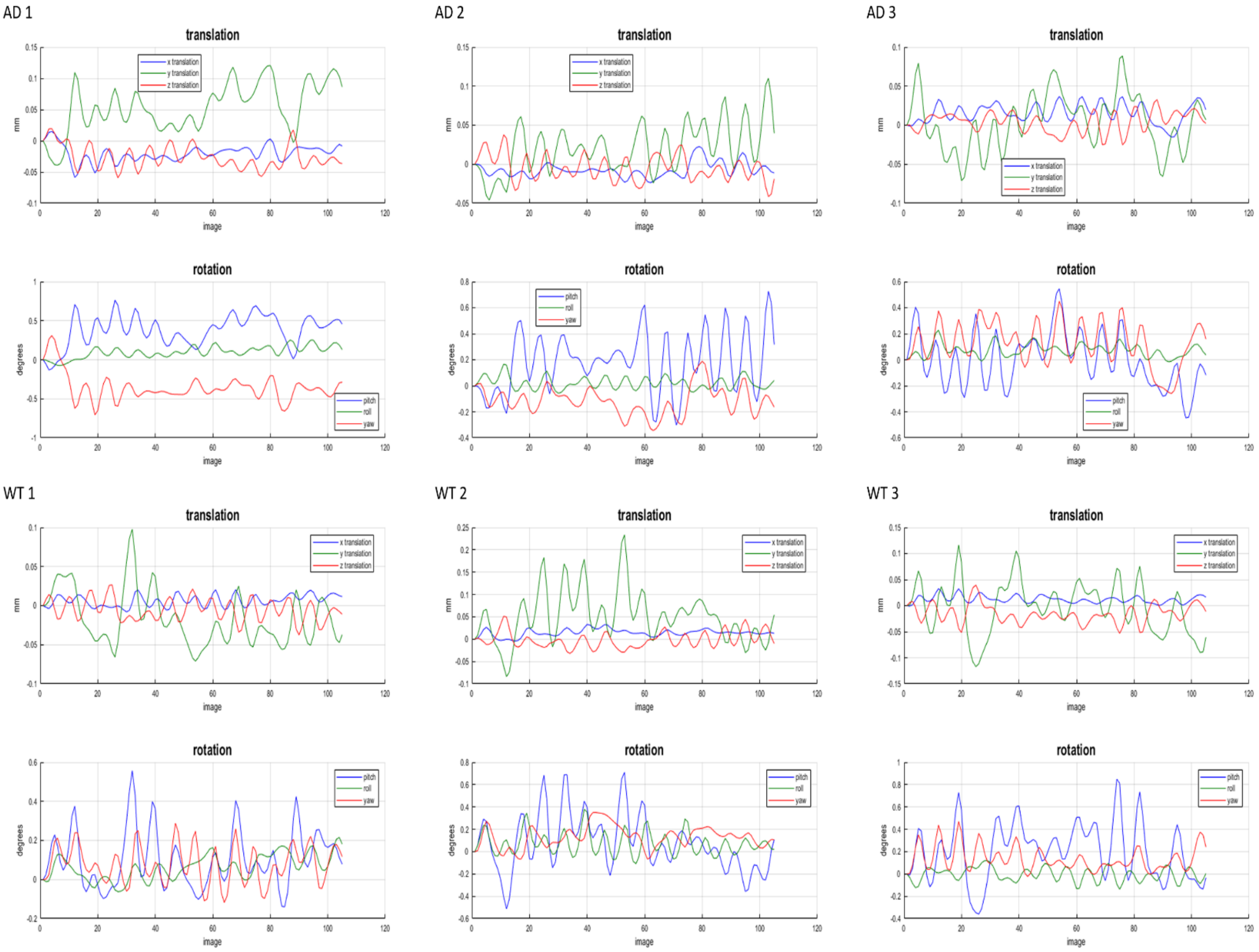

Supplementary Figure 6. Motion detection during the fMRI data acquisition
Data on head motion during the fMRI session for all mice are shown.
The horizontal axis of the translation figures represents the number of image volumes, and the vertical axis represents the moving distance (mm). The blue line (x translation) represents moving distance of sagittal plane. The green line (y translation) represents moving distance of transverse plane. The red line (z translation) represents moving distance of coronal plane.
The horizontal axis of the rotation figures represents the number of image volumes, and the vertical axis represents rotated angle (°). The blue line represents rotation of pitch. The green line represents rotation of yaw. The red line represents rotation of roll.
